# Validation of remote height and weight assessment in a rural randomized clinical trial

**DOI:** 10.1186/s12874-022-01669-8

**Published:** 2022-07-11

**Authors:** Bethany Forseth, Ann M. Davis, Dana M. Bakula, Megan Murray, Kelsey Dean, Rebecca E. Swinburne Romine, Kandace Fleming

**Affiliations:** 1 Center for Children’s Healthy Lifestyles & Nutrition, Kansas City, MO USA; 2 Department of Pediatrics, University of Kansas Medical Center, 3901 Rainbow Boulevard, CDU 2036, Kansas City, USA; 3 Department of Pediatrics, Children’s Mercy Kansas City, Kansas City, MO USA; 4 Life Span Institute, University of Kansas, Lawrence, Kansas, USA

**Keywords:** Height, Weight, Remote assessment, Clinical trial, Rural, Body mass index

## Abstract

**Background:**

The purpose of this study is to describe and assess a remote height and weight protocol that was developed for an ongoing trial conducted during the SARS COV-2 pandemic.

**Methods:**

Thirty-eight rural families (children 8.3 ± 0.7 years; 68% female; and caregivers 38.2 ± 6.1 years) were provided detailed instructions on how to measure height and weight. Families obtained measures via remote data collection (caregiver weight, child height and weight) and also by trained staff. Differences between data collection methods were examined.

**Results:**

Per absolute mean difference analyses, slightly larger differences were found for child weight (0.21 ± 0.21 kg), child height (1.53 ± 1.29 cm), and caregiver weight (0.48 ± 0.42 kg) between school and home measurements. Both analyses indicate differences had only minor impact on child BMI percentile (− 0.12, 0.68) and parent BMI (0.05, 0.13). Intraclass coefficients ranged from 0.98 to 1.00 indicating that almost all of the variance was due to between person differences and not measurement differences within a person.

**Conclusion:**

Results suggest that remote height and weight collection is feasible for caregivers and children and that there are minimal differences in the various measurement methods studied here when assessing group differences. These differences did not have clinically meaningful impacts on BMI. This is promising for the use of remote height and weight measurement in clinical trials, especially for hard-to reach-populations.

**Trial registration:**

Clinical. Registered in clinicaltrials.gov (NCT03304249) on 06/10/2017.

## Background

The outbreak of the SARS COV-2 virus has had significant impacts on ongoing research, especially research that involved contact with patients [1]. Many clinical trials were halted in the spring of 2020 due to concerns about infection [2]. Some clinical trials were able to resume after changes to study protocols to protect patient safety while also achieving their stated scientific aims. Many of these changes involved moving to remote data collection.

Height and weight are common measures obtained as part of clinically oriented research, yet very little is known about obtaining height and weight remotely. Objective measures collected by study staff using standard research-grade equipment are preferred over self-report methods as they are less subject to bias and are more accurate [3–10]. One solution for collecting these measures remotely is to have participants weigh themselves on a scale provided by the study team, or to use an existing home scale, and then report their weight to study staff.

Remotely collecting height and weight has proven to be feasible. Studies examining weighing protocols where participants take their weight at home using e-scales support feasibility and concordance in measures compared to clinic staff [11, 12], in adult populations. E-scales also remove potential reporting bias by participants by transmitting data directly to researchers/clinicians. While there are many benefits to using e-scales, their technology requirements (smart phone, app, internet connection) may be prohibitive to integration in large research studies or for certain populations (such as rural individuals). Studies using regular scales (e.g., not smart scales or e-scales) and stadiometers also support feasibility of protocols but demonstrate potential room for bias and more detailed instructions for the measurements are required. For instance, Paez and colleagues (2014) recruited 30 women from a larger study to self-report their height (without measuring) and to report their weight from scales provided by the study [13]. Research staff provided participants with a weighing protocol via printed instructions and through an instructional phone call. Within 2 weeks of participants reporting their weight, researchers visited the homes of a subset of these participants to remeasure their weight using a scale of the same model and obtain a height measure. Self- and researcher-measured weight had an overall mean difference of 0.93 ± 0.27 kg. Additionally, self-reported height differed from researcher-measured height by an overall mean difference of 0.56 ± 1.91 cm. More recently, Ghosh-Dastidar et al. [14] conducted an in-lab study where the research team led participants through a standard protocol to collect their own height and weight via video-conferencing, then immediately after this, height and weight were checked by a trained research team member. This study observed small overall mean differences between participant and researcher-reported data (weight differed by 0.04 ± 0.09 kg; height differed by 0.11 ± 0.02 cm); these differences were not determined to be clinically meaningful.

There may be additional considerations when conducting remote height and weight collection with children and adolescents. Specifically, when measuring child height and/or weight remotely, caregivers will typically be responsible for the measurement, and there may be additional biases that impact how caregivers report a child’s height and/or weight. In a recent study of adolescents with overweight or obesity, data from smart scales demonstrated that participants were less likely to report their weight if it was a higher value compared to previous days [15]. Another study observed that caregivers of children with overweight/obesity under-reported their child’s height measurements resulting in a difference of 0.86 cm compared to a difference of 0.1 cm for children in the normal weight category. Despite these differences in height measurements, caregivers reported comparable weight measurements [16] across all weight classifications. It remains unclear how accurate remote height or weight measurements are for school-aged children.

Although these studies indicate that it may be possible to obtain height and weight remotely, more applied examples of remote validation of height and weight measures are needed in real-world clinical trials, especially clinical trials with pediatric patients. Therefore, the aim of the current paper is to describe the implementation of a fully remote height and weight collection protocol as part of a family-based healthy lifestyle clinical trial for underserved, rural children with overweight or obesity and their caregivers. Additionally, we aim to describe the discrepancies observed in height and weight among both adults and children, as well as assess factors that may have impacted the validity of the home height and weight measurements.

## Methods

### Sample

The study sample was drawn from participants enrolled in the iAmHealthy Schools trial (NCT03304249 in clinicaltrials.gov on 06/10/2017) [17], which is a cluster-randomized study that tests the iAmHealthy lifestyles program in comparison to a newsletter control in rural 2nd thru 4th grade children (NIH R01 NR016255). Children (8.3 ± 0.7 years; 66% female) with overweight/obesity (body mass index percentile [BMI%ile] ≥85th) and their primary caregivers (38.2 ± 6.1 years; 92% female) who were attending one of 18 participating rural elementary schools in a single midwestern state were invited to participate. The intervention and study methods are reported elsewhere [14].

### Procedure

**Fig. 1:**
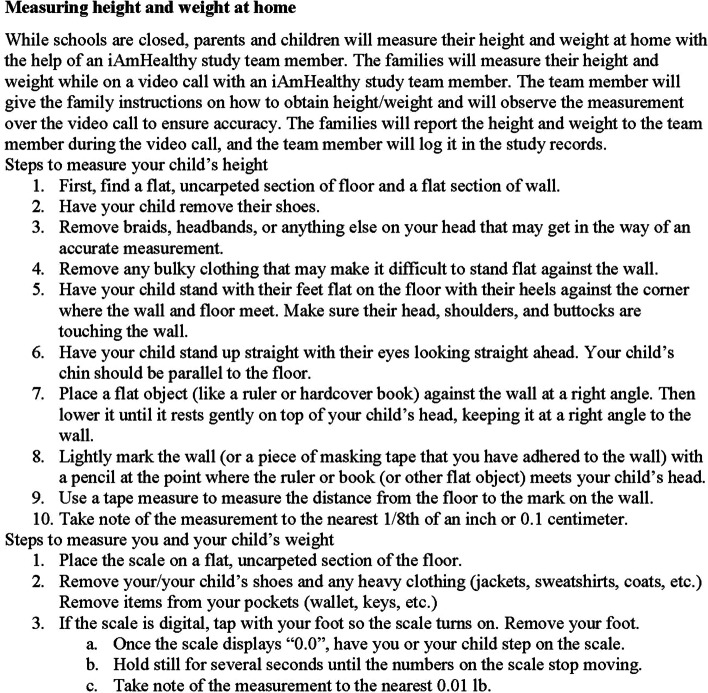
Written instructions given to participating families

Per the initial study protocol [17], child height and weight and caregiver weight were to be taken at their local elementary school by a fully trained school staff member on research grade equipment provided by the study at three primary timepoints: baseline, 8 months (post-treatment), and 20 months later (long-term follow-up) as well as monthly during the initial 8-month period. Caregiver height was measured at baseline and this height measure was used for the remainder of the study. In March of 2020, the SARS COV-2 pandemic began to impact school schedules and resulted in the closure of school buildings. As the intervention for the study was delivered fully remotely via interactive televideo, the decision was quickly made to transition the collection of height and weight to be fully remote as well. This decision allowed the clinical trial to continue as scheduled and largely as planned during the time of the pandemic. For the height and weight measures during school closures, families chose whether to use existing equipment in the home or use new equipment mailed to them by the study. Written (Fig. 1) and video instructions were developed and shared (https://www.youtube.com/watch?v=kKcKodvYBnk&feature=youtu.be) with the participating families.

Between September 2020 and February 2021, participating families from 5 of the 18 schools in the trial were invited to participate in the current sub-project to validate the newly developed home height/weight procedures for their 8-month or 20-month measures, depending upon where they were in the protocol timeline. Baseline data were previously collected per protocol for each of these families, prior to implementation of the remote home height/weight procedures [17]. All measurements taken at the school were obtained by the trained school staff. All study procedures were approved by and followed local IRB guidelines and regulations. Participant consent and/or assent were obtained prior to data collection.

### Measures

#### Weight

School-scale weights were taken on a portable SECA digital scale, Model 813 (SECA, Hamburg, Germany; 440 pounds) which is accurate within 0.1 pounds over a range from 1 to 440 pounds [16]. Home scale weights were taken on an existing home scale (models not noted) or on a scale provided by the research team that was widely available during the time of the pandemic despite supply chain and shipping issues experienced at that time (Etekcity High Precision Digital Body Weight Bathroom Scale with Ultra-Wide Platform and Easy-to-Read Backlit LCD; 440 Pounds). All weights were taken with light clothing and no shoes in triplicate and recorded to the nearest 0.1 lb. (for home weights) or 0.1 kg (for school weights); mean values were used in analyses. To compare the home scale to the school scale, each family was asked to bring their existing home scale or research-provided scale to the school during a school measurement session, and the two scales (one brought from home and one at the school) were used to obtain measures during the same session.

#### Height

Child home height was measured by their caregiver using an existing tape measure in the home (models not noted) or using a tape measure provided by the research team (AmazonBasics Heavy Duty Tape Measure, 16 ft). Child home height was measured at home prior to the school measurement session, within 72 hours; if caregivers experienced difficulty following the instructions in Fig. 1, they were instructed to contact study staff. Child school height was measured on a SECA stadiometer, Model 213 (SECA, Hamburg, Germany). During height measurements, participants were instructed to remove shoes, stand against the wall and look straight ahead, following the detailed procedures outlined in Fig. 1. Height was taken in triplicate and recorded to the nearest 0.1 cm; mean of the three measures was used in analyses. Caregiver height was measured at the baseline period by trained school staff.

#### Body mass index

Researchers calculated body mass index (BMI) for caregivers from the height and weight data, using standard calculations. Body mass index percentile (BMI%ile) for children was calculated per sex and age standard equations from the CDC [18].

#### Demographic information

Caregivers completed surveys at baseline regarding demographic characteristics including child and caregiver sex and race/ethnicity, caregiver education, marital status, household income and eligibility for free or reduced lunch.

### Analyses

To compare these findings to previous research in this area, analyses include overall mean difference. However, because the moderate over and under estimation offset each other when examining the overall mean difference, analyses focused on examining the absolute amount of difference between the two types of measurements (absolute mean difference). Analyses were also conducted to identify factors which may have impacted the discrepancies between school and home measurements.

#### Absolute mean difference

Mean values were calculated for the three measurements taken for each person comparing home scale and school scale for child and parent weight and comparing child home height and child school height for child height. The mean and standard deviation of these mean values for child and parent weight, child height, BMI%ile for children, and BMI for caregivers were computed for each approach. Next, one-sample t-tests were used to determine if the mean absolute value of the difference between measurements was significantly different from 0. This test enabled us to quantify the extent to which the two measurements differed from each other ignoring sign, which has implications for making clinical decisions about individuals.

#### Overall mean difference

We used paired samples t-tests to examine within-person differences between school scale/home scale for child and parent weight, and for child home height and child school height for child height measurements. These tests enabled us to determine if there was a systematic difference wherein one measure was consistently higher than the other. The average within-person difference and the standard deviation of that difference are reported. Agreement was further investigated by examining Bland-Altman plots [19] of school differences versus home measurement with 95% limits of agreement (LOA). Intraclass correlation coefficients were calculated between the school and home measurements of height and weight to enable us to determine how much of the variance in schools was due to measurement.

#### Exploratory analyses

Analyses were conducted to identify factors which may have impacted the discrepancies between measurements. Factors evaluated include number of days between the two measurements, caregiver education, and home scale weight taken using existing equipment in the home vs. home scale weight taken on a scale provided by the research team.

## Results

Of the 141 families from the 18 schools in the larger clinical trial, 48 families from 5 schools were invited to take part in the current validation study; 38 of these families participated (79.17% participation rate). Rural child and caregiver participants were primarily white, and caregivers were largely college-educated and married (see Table 1), representative of the overall sample and the geographic area from which participants were recruited. Of those who participated, 22 families (58%) chose to have the research team provide them a scale, and 20 families (53%) chose to have the study send them a tape measure. Sample size varied across measurements (see Table 2).

**Table 1:** Characteristics of families who obtained height and weight measurements

Characteristics	n (%)
**Child Characteristics**	
Sex	
Female	25 (65.8)
Ethnicity	
Hispanic/Latino	7 (18.4)
Race	
White	32 (84.2)
> 1 race reported	5 (13.2)
Native American	1 (2.6)
Child eligible for free/reduced lunch	20 (52.6)
**Caregiver Characteristics**	**n (%)**
Sex	
Female	27 (81.8)
Ethnicity	
Hispanic/Latino	2 (6.1)
Race	
White	25 (71.4)
> 1 race reported	4 (11.4)
Native American	1 (2.9)
Missing	5 (14.3)
Education	
≤ High school degree	6 (15.4)
Some college, no degree	10 (25.6)
Vocational or college degree	17 (43.6)
Graduate degree	5 (12.8)
Missing	1 (2.6)
Marital Status	
Single	6 (15.4)
Married	24 (61.5)
Separated	1 (2.6)
Divorced	5 (12.8)
Other/Missing	3 (7.7)
Income	
< $50,000	19 (50.0)
$50,000 – $99,999	15 (39.5)
≥ $100,000	4 (10.5)

**Table 2:** Comparison of home measurements and school measurements

	Sample	Home	School	Overall Mean Diff. (School-Home)	Absolute Mean Diff
M (SD)					
Weight (kg)	Child *n* = 23	54.72 (14.31)	54.82 (14.34)	−0.10 (0.28)	0.21 (0.21)
	Caregiver *n* = 32	108.18 (26.75)	107.96 (26.81)	−0.22 (0.61)	0.48 (.42)
Height (cm)	Child *n* = 34	146.01 (9.44)	146.27 (9.65)	0.26 (2.00)	1.53 (1.29)
BMI%ile	Child *n* = 20	96.33 (4.97)	96.21 (5.39)	−0.12 (1.55)	.68 (1.39)
BMI	Caregiver *n* = 32	39.51 (10.19)	39.46 (10.24)	−0.05 (0.15)	0.13 (0.10)

*M* mean, *SD* Standard Deviation

### Weight measurements

Of the participants in this study, 87% of child measurements and 97% of caregiver weight measurements were taken at the school, comparing school scale to home scale in the same measurement session. Three families were unable to bring in their home scale to the school measurement session and were therefore asked to self-report their weight once they returned home, based upon a new measurement on their home scale (using procedures in Fig. 1). For children, the average absolute mean difference between school scale weight and home scale weight was 0.21 kg (Table 2). This absolute mean difference was statistically significantly different from zero [t(22) = 4.63, *p* < 0.001, d = 0.97] but in terms of clinical significance was less than the reported standard errors from other studies (0.45 ± 0.8 kg) [18]. Sixty-five percent of the home measurements for children were within 0.2 kg of the school measurement and 87% were within 0.5 kg of the school measurement. For caregivers, the absolute mean difference between school scale weight and home scale weight was 0.48 kg. This was also statistically significantly different from zero [t(31) = 6.52, *p* < 0.001), d = 1.15], but in terms of clinical significance was well within differences (0.93 ± 0.27 kg) reported in previous work [11]. Forty-three percent of the home measurements for caregivers were within 0.2 kg of the school measurement and 67% were within 0.5 kg of the school measurement.

Regarding the overall mean difference in weight for the children (Table 2), the mean for the home scale weight was 0.10 kg higher than the mean for the school scale weight on average. This was not a statistically significant difference [t(22) = − 1.73, *p* = 0.10, d = .36]. Regarding the overall mean difference in weight for the caregivers, the mean home scale weight was 0.22 kg higher than the mean school scale weight on average. This difference was approaching statistical significance [t(31) = − 2.04, *p* = 0.05, d = .36].

**Fig. 2:**
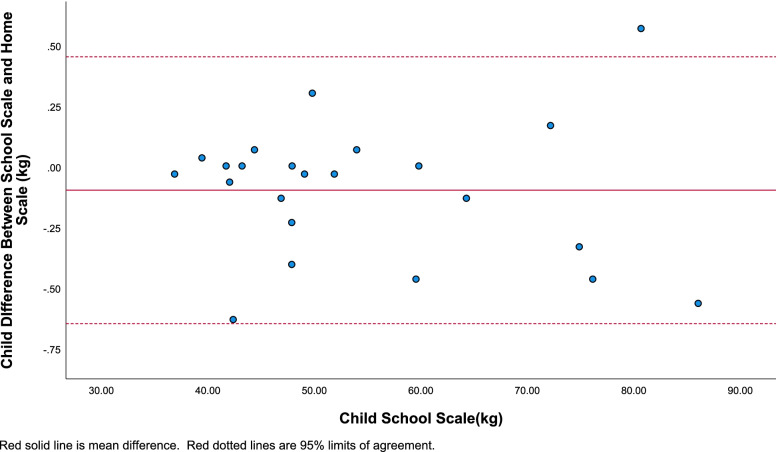
Bland-Altman plot for child school scale measurement compared to the difference between child school scale and home scale weight

**Fig. 3:**
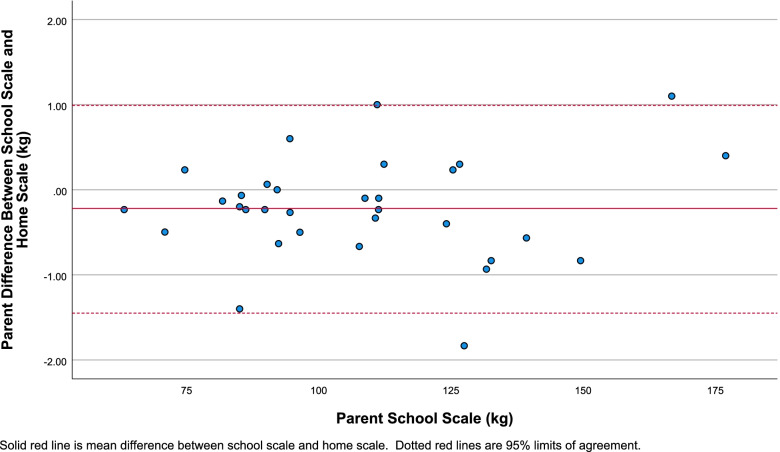
Bland-Altman plot for parent school scale measurement compared to the difference between parent school scale and home scale weight

Weight differences for adults and children are reported in Bland-Altman plots (Figs. 2 and 3), which provide an indication of discrepancy in school scale weight measurements relative to home scale weight measurements. The difference between school scale and home scale weight measurements is plotted on the y-axis with the school scale weight measurement on the x-axis. The middle red line represents the mean difference between scale measurements. The dashed lines above and below represent the 95% limits of agreement (LOA). The mean differences for child and caregiver weight indicated moderate over estimation by home scale weight.

#### Factors impacting discrepancies in weight

*Measurement type*. Intraclass correlation coefficients were calculated between school scale and home scale weight measurements for both children and caregivers and for height measurements for children between child home height and child school height. Coefficients ranged from 0.98 to 1.00 indicating that very little variance in measurements was due to the measurement type. *Number of days between measurements - children.* The impact of number of days between the two weight measurements was examined. For children, almost all participants were weighed on the same day on the home scale and the school-scale; one child was measured with a one-day separation. The difference in this child’s weight was 0.41 kg which is within one and a half standard deviations of the mean difference between measurements. There were five children whose weight was assessed on the same day that were more discrepant than this one weight that was on different days. Thus, the weight difference with a time delay for children was not extreme within the distribution of all discrepancies. *Number of days between measurements – caregivers.* Regarding caregiver weights, two adults were weighed with a lag between measurements. For the first, measures were separated by 3 days and the weight difference was 0.27 kg. For the second, the measures were separated by 2 days and the difference was 0.67 kg. There were eight participants who were measured on the same day with larger differences than 0.67 kg, which was within one standard deviation of the average discrepancy. Thus, the weight differences with a time delay for adults were well within the distribution of discrepancies. *Existing home scale* vs. *research provided home scale.* Next, the impact of using an existing home scale compared to using a newly purchased research provided home scale was assessed. Results indicated a greater tendency for participants using new research-provided home scales (as opposed to existing home scales) to over-estimate weight as compared to measurements obtained on school scales. For children, measurements on new research scales overestimated by an average of 0.18 kg, which was statistically significantly different from 0, *p* = 0.02. Measurements of children on existing home scales were under-estimated by 0.03 kg on average, which was not significantly different from zero. Similarly, measurements of parent weight on new research-provided scales were overestimated by 0.32 kg on average, which was significantly different from 0, *p* = 0.002. While parent measurements on existing home scales were also overestimated compared to measurements obtained on school scales by an average of 0.02 kg, this was not statistically significantly overestimation.

### Height discrepancies

**Fig. 4:**
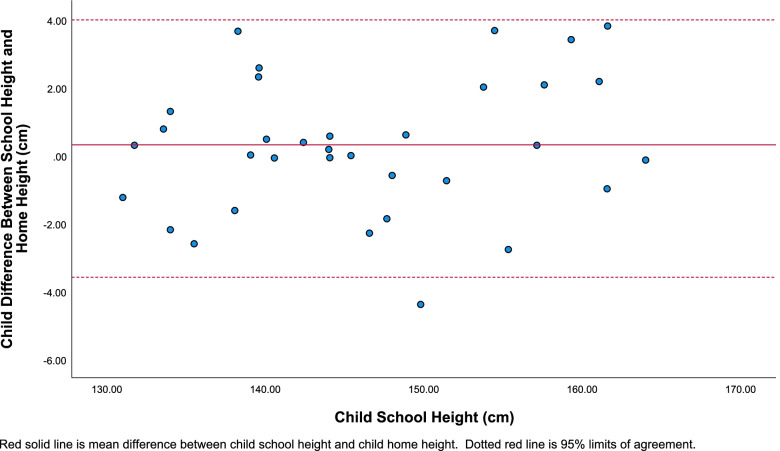
Bland-Altman plot for child school height measurement compared to the difference between child school height and home height measurements

The absolute mean difference in child home height child school height was 1.53 cm (Table 2). This was statistically significantly different from 0 [t(33) = 6.93, *p* < 0.001, d = 1.19]. For children, the mean school height was 0.26 cm higher than the mean home height. This was not a statistically significant difference [t(33) = 0.74, *p* = 0.46, d = 0.13]. Height differences for children are reported in a Bland-Altman plot (Fig. 4). The plot shows moderate over and under estimation across levels of school height.

#### Factors impacting discrepancies in height

Number of days between the two measurements for children was examined. Lag between home and school height measurements ranged from 0 to 33 days with 29% of observations occurring the same day and 50% occurring within 3 days. Days between observations and absolute difference in height measurement were not correlated (*r* = − 0.10, *p* = 0.58), indicating no significant relationship between lag and height difference. Height for caregivers was only obtained at baseline, and all baseline measures were completed prior to the current validation study.

### Body mass index discrepancy

The absolute mean difference in BMI%ile between the “home” measurements and the “school” measurements was 0.68. This difference was statistically significantly different from zero [t (19) = 2.19, *p* = 0.042, d = 0.49]. For children, the Body Mass Index percentile (BMI%ile) obtained by “home” measures was 0.12 higher than the percentile based on “school” measures. This was not a statistically significant difference [t (19) = − 0.35, *p* = 0.73, d = 0.08].

#### Factors impacting discrepancies in child body mass index percentile

Child BMI%ile assessed with research-provided scales overestimated by an average of 0.28 points. Similarly, child BMI%ile assessed with an existing home scale overestimated by an average of 0.61 points. Neither overestimate was statistically significant (*p* = 0.52 and *p* = 0.30, respectively). The absolute mean difference in BMI%ile scores was not significantly correlated with caregiver education (*r* = − 0.26, *n* = 20, *p* = 0.26) or with number of lag days between the two height measurements (*r* = − 0.07, *n* = 20, *p* = 0.76).

#### Factors impacting discrepancies in caregiver body mass index

The absolute mean difference in parent BMI between the home scale weight and the school scale weight (both using the baseline height) was 0.13 kg/m^2^. This difference was statistically significantly different from zero [t(31) = 7.33, *p* < 0.001, d = 1.30]. The average home scale parent BMI was 0.05 kg/m^2^ higher than school scale parent BMI. This difference was not statistically significantly different from zero [t(31) = 1.85, *p* = 0.07, d = 0.33]. We then assessed the impact of scale type (existing home scale, research-provided home scale); parents using the research-provided home scale overestimated their BMI significantly by 0.08 kg/m^2^ on average, *p* = 0.002, while parents using existing home scales obtained the same average BMI as that measured by trained staff using research grade equipment. The absolute value of the difference in BMI measurement was not significantly correlated with caregiver education (*r* = 0.21, *n* = 31, *p* = 0.27), indicating that those who have more education (e.g., college degree) performed similarly to those with less education (e.g., high school or less).

## Discussion

Measurement of height and weight of caregivers and children is applicable to many clinical trials, including those impacted by the SARS COV-2 pandemic. Our group was conducting a remote family-based healthy lifestyle intervention for underserved rural children with overweight and obesity prior to the beginning of the pandemic, but our protocol included in person height and weight measurements for children and parents at the local rural elementary school using research-grade equipment. Above we describe the highly feasible procedures used by our team for transitioning to fully remote measurement with widely available equipment, which were highly feasible for our study and potentially useful for future studies.

Overall mean difference analyses indicate small and insignificant differences between the assessment methods studied, which is clearly demonstrated in the Bland-Altman plots. The obtained differences were minimal and similar in magnitude to those obtained in previous studies [13–15]. In children, the differences in weight measurements were slightly smaller and height measurements were slightly larger compared to results found by Tenenbaum et al.; they observed differences of 0.45 ± 0.8 kg and 0.1 ± 1.3 cm between home and clinic measures when the assessments were obtained 1 day apart [16]. Similarly, the differences in weight measurements for caregivers were slightly lower compared to three of the four previous studies (range of weight differences observed in previous studies was 0.6–1.1 kg) [11–13]. Only Ghosh-Dastidar et al., reported smaller mean differences in weight (0.02 ± 0.4 kg) when compared to our study [14]. Overall, these findings indicate negligible differences in BMI between measurement methods which would likely not have a systematic or meaningful impact on BMI in trial results.

To add to the literature, our study also conducted analyses using the absolute mean difference statistic. These types of analyses are important to understand how variation in measurement methods may impact the results of a single individual over time, rather than of a group of participants over time. The absolute mean difference analyses conducted here, specifically for child BMI%ile and adult BMI, were statistically significant, implying that varying equipment/methods across measurements may have a significant impact on findings for a single individual. This extends the literature in this area and to our knowledge is the first time that absolute mean difference has been applied in a measurement validation paper of this type. We also evaluated several factors which may have contributed to the lack of agreement between measurements (lag time between measures, home-supplied scale vs. researcher supplied scale, caregiver education). Analyses indicate that the only factor that had a significant impact on the measurements was a research-provided scale; specifically, the research provided scales seemed to overestimate weight to a greater degree than existing home scales when each was compared to the research grade school scale. Although home and research scales were not calibrated in the current study, future research may want to assess whether the lack of calibration contributed to differences or whether there are other reasons for these differences (such as lack of voiding prior to measurement, or time of day of measurement).

This study has several strengths, including that it is the first to evaluate the accuracy of remote height and weight measurement procedures among school-age children. Second, the equipment used was highly practical (inexpensive and purchased from an online web vendor with free delivery to participant homes; not requiring internet in the home or a smart phone; minimal set up), thus potentially making our procedures more applicable for other clinical trials in ‘real-world’ remote settings. Finally, our use of absolute mean difference analyses is new to the field and has implications for studies considering this type of methodology or clinicians who may want to implement similar procedures. Limitations within the study include the predominantly white, rural sample and the age range of elementary school children and their parents. These results may not be generalizable for the assessments of infants, adolescents, or children from different backgrounds. Also, the research provided home scales were not e-scales; future researchers may wish to study the feasibility of using these types of e-scales with participants as the findings may be different than those obtained here. Additionally, we were not able to account for time-of-day differences taken between home and school measurements, although some studies indicate time-of-day may not have meaningful impact on these measures [11]. Finally, we did not include a measure of caregiver fidelity to the at-home collection protocol, which is an area of planned future research.

## Conclusion

In sum, these data suggest that these height and weight measurements by families of school-aged children enrolled in clinical trials is not only feasible but is relatively accurate. Remote collection of height and weight may increase the feasibility of conducting research with rural and other underserved populations who are unable to travel for study visits as well as conducting research during pandemics in which physical distancing is critical for public health and safety. Finally, these results suggest that remote height and weight collection can be executed at a low cost, as existing home scales were largely accurate, and the materials sent to participants (who did not have their own materials) were low cost readily available to ship and required little to no set up. Overall, these results are promising for the potential to continue to rely on remote height/weight measurement in weight management clinical trials with school-aged children.

## Data Availability

The datasets used and/or analysed during the current study are available from the corresponding author on reasonable request.

## Abbreviations

BMI: Body mass index
BMI%ile: Body mass index percentile
